# Device and circuit-level performance of carbon nanotube field-effect transistor with benchmarking against a nano-MOSFET

**DOI:** 10.1186/1556-276X-7-467

**Published:** 2012-08-19

**Authors:** Michael Loong Peng Tan, Georgios Lentaris, Gehan Amaratunga AJ

**Affiliations:** 1 Faculty of Electrical Engineering, Universiti Teknologi Malaysia, UTM Skudai, Johor, 81310, Malaysia; 2 Electrical Engineering Division, University of Cambridge, 9 J.J. Thomson Ave, Cambridge, CB3 0FA, UK

**Keywords:** Device modeling, HSPICE, Benchmarking, MOSFET, CNTFET, Logic gates

## Abstract

The performance of a semiconducting carbon nanotube (CNT) is assessed and tabulated for parameters against those of a metal-oxide-semiconductor field-effect transistor (MOSFET). Both CNT and MOSFET models considered agree well with the trends in the available experimental data. The results obtained show that nanotubes can significantly reduce the drain-induced barrier lowering effect and subthreshold swing in silicon channel replacement while sustaining smaller channel area at higher current density. Performance metrics of both devices such as current drive strength, current on-off ratio (*I*_on_/*I*_off_), energy-delay product, and power-delay product for logic gates, namely NAND and NOR, are presented. Design rules used for carbon nanotube field-effect transistors (CNTFETs) are compatible with the 45-nm MOSFET technology. The parasitics associated with interconnects are also incorporated in the model. Interconnects can affect the propagation delay in a CNTFET. Smaller length interconnects result in higher cutoff frequency.

## Background

Carbon nanotubes (CNTs) have been proposed as an alternative channel material to silicon (Si), based on their quantum transport properties which, in principle, allow ballistic transport at room temperature. CNT ballistic modeling [1] has been used to assess the performance of the device at the HSPICE circuit level [2]. Device modeling is vital for projecting the practical performance of a CNT transistor as a switching device in integrated circuits (ICs).

We report the potential of a CNT channel through modeling as a substitute to a silicon channel in a scaled metal-oxide-semiconductor field-effect transistor (MOSFET) for logic applications. By scaling the Si transistor and the density of states (DOS) of the CNT, we observe good agreement between CNT and ballistic Si MOSFET [3] in the drain current–voltage (*IV*) output characteristics. Output current is critical in determining the switching speed of a transistor in logic gates. We show that the output performances of CNT and Si channel devices are similar in the 45-nm node experimental data. However, the modeling results point to significant reduction in drain-induced barrier lowering (DIBL) and related high field effects in the CNT compared to the short-channel nanoscale Si MOSFET at the same output current. We also assess the effect of channel area restructuring on electric field properties as well as the role of the DOS in determining CNT current. Unlike in the Si MOSFET, it is seen that the performance of a CNT channel is enhanced when the source/drain width is minimized rather than the channel length due to gate-to-source/drain parasitic fringe capacitances. MOSFET scaling according to Moore's law is limited by process controllability.

## Methods

### Carbon nanotube and MOSFET modeling

**Figure 1:**
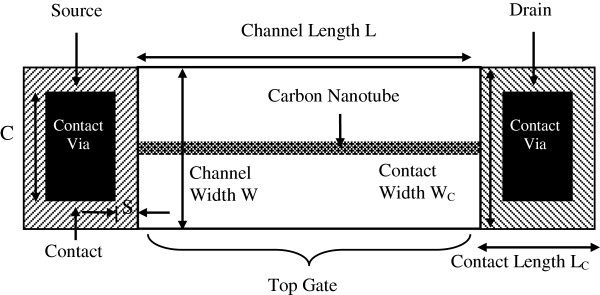
Top view of CNTFET device.

A layout of a carbon nanotube field-effect transistor (CNTFET) is shown in Figure 1. The area of the channel is defined by the width (*W*) of the source and drain contacts and the length (*L*) of the nanotube. Details of the ballistic MOSFET modeling can be found in our previous work [3].

**Figure 2:**
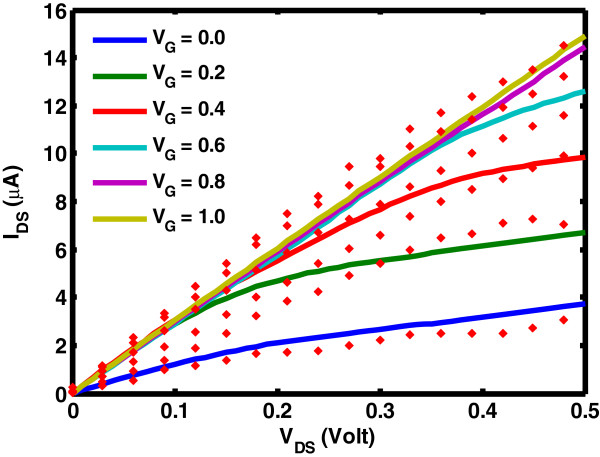
**Simulated CNT drain characteristic versus 80-nm experimental data.** Simulated single-subband CNT drain characteristic (solid lines) versus 80-nm experimental data with high-potassium (K)-doped source and drain doping (filled diamond) at *V*_G_ = 0 to 1.0 V in 0.2-V steps. (Adapted from [9]).

The analytical carbon nanotube model comes from the work of Rahman et al. [4,5] where we have extended the universal DOS spectral function into a numerical calculation for CNT conduction subbands. We have modified the DOS subroutine [6] to account for multimode transport [7]. To improve precision and accuracy in the simulation, the parameters in Table 1 for MOSFET and CNTFET which incorporate quasi-ballistic transport scattering are extracted from CADENCE [8] and Javey et al. [9], respectively. CNTFET analytical models have been validated and agree well with experimental data [9,10] particularly in the saturation region depicted in Figure 2.

**Table 1:** Source and drain capacitance for multiple substrate insulator thickness

**Substrate insulator thickness (nm)**	** *C* **_**sb**_**or *C* **_**db**_**(aF)**	** *I* **_**ds**_**(μA) at *V* **_**G**_ **= 1 V**
10	34.53	47.395
50	6.906	47.340
100	3.453	47.272
200	1.727	47.135
300	1.151	46.998
400	0.863	46.860
500	0.691	46.723

If a CNT can achieve the same current as a MOSFET, an identical channel area (*A*_MOS_ = *A*_CNT_) can be maintained by setting the width of the physical space occupied by the CNTFET to be *W*_CNT_ = *A*_MOS_ / *L*_CNT_. When *W* = *L* for the MOSFET, the general channel area can be expressed as *A* = (*kL*)^2^, where *k* is the scaling factor. As such, a CNT channel with length, 2*kL* should attain the same current with *W* = 0.5*kL*. Thus, if the physical width of the CNT channel is *W* ≤ 0.5*kL*, there will not be any area drawback in output current due to the longer *L*. In fact, the maximum electric field in CNT is halved, giving *E*_mCNT_ = *E*_mSi_ / 2, and is significantly reduced as the CNT channel grows longer. For a CNT with *L* = 60 nm compared to a Si MOSFET with *L* = 45 nm, the maximum electric field is *E*_m_ = 0.83 *E*_mSi_.

### Device modeling

**Figure 3:**
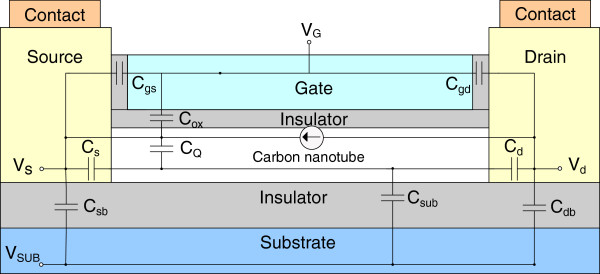
HSPICE macromodel for CNTFET.

The top view of CNTFET with the source and drain contacts is shown in Figure 1. The filled black rectangle represents the contact enclosure with dimension extracted from a generic 45-nm MOSFET process design kit (PDK) where *S* = 20 nm, *C* = 60 nm, and *W*_C_ = *L*_C_ = 100 nm. Nine capacitances are introduced into the carbon-based macromodel as illustrated in Figure 3. They are the gate oxide capacitance *C*_ox_, quantum capacitance *C*_Q_, source capacitance *C*_s_, drain capacitance C_d_, substrate capacitance *C*_sub_, source-to-bulk capacitance *C*_sb_, drain-to-bulk capacitance *C*_db_, gate-to-source capacitance *C*_gs_, and gate-to-drain capacitance *C*_gd_. The size of the contact is crucial as it ultimately influences *C*_sb_ and *C*_db_. They are given in Table 1 and can be written as

(1) $$C_{\text{sb}}\;\text{or}\;C_{\text{db}}=\varepsilon_{\text{ins}}\left(\frac{WL}{t_{\text{ins}}}\right)\text{,}$$

where *t*_ins_ is the thickness of the insulator, *W* is the width of the contact, *L* is the length of the contact, and *ε*_ins_ is the permittivity of the insulator. The substrate insulator capacitance *C*_sub_ for CNTFET is given by

(2) $$C_{\text{sub}\_\text{CNTFET}}=\frac{2\pi \varepsilon_{\mathrm{ins}}}{\ln\left(\frac{4t_{\mathrm{sub}}}{d}\right)}\text{,}$$

where *t*_sub_ is the substrate oxide thickness and *d* is the diameter of CNT. The intrinsic gate capacitance *C*_G_ of CNTFET is a series combination of gate oxide capacitance *C*_ox_ and quantum capacitance *C*_Q_[11]. The *C*_ox_ of a CNTFET [12-14] is shown to be

(3) $$\text{Nanotube}\;C_{\text{ox}}=\frac{2\pi \varepsilon_{\text{ins}}}{\ln\left(\frac{2t_{\text{ins}}+d}{d}\right)}$$

The quantum capacitance is expressed by [15-17]

(4) $$C_{\text{Q}}=\frac{2g_{\text{v}}g_{\text{s}}q^{2}}{hv_{\text{F}}}\sum_{i}\frac{E}{\sqrt{E^{2}-{\left(E_{\text{Gi}}/2\right)}^{2}}}\;\Theta \left(\left|E\right|-\frac{E_{\text{Gi}}}{2}\right)\text{,}$$

where *g*_s_ is the spin degeneracy, *g*_v_ is the valley degeneracy, *E*_Gi_ is the bandgap energy, and *v*_F_ is the Fermi velocity. The step function $\Theta \left(x\right)$ is equal to 1 when *x* > 0 and 0 when *x* < 0. The *C*_gs_ and *C*_gd_ are given as

(5) $$C_{\text{gs}}=\frac{L_{\text{g}}C_{\text{ox}}}{2}\left[\frac{C_{\text{Q}}+C_{\text{s}}}{C_{\text{tot}}+C_{\text{Q}}}\right]\text{;}$$

(6) $$C_{\text{gd}}=\frac{L_{\text{g}}}{2}C_{\text{ox}}\left[\frac{C_{\text{Q}}+C_{\text{d}}}{C_{\text{tot}}+C_{\text{Q}}}\right]\text{,}$$

where *C*_s_ and *C*_d_ are the source and drain capacitance fitting parameters, respectively, [1,2] that are used to fit the experimental data and *L*_g_ is the length of the gate. The sum of *C*_gd_ and *C*_db_ gives the intrinsic capacitance *C*_int_.

The square law is no longer valid for *I-V* formulation of short-channel MOSFET. Tan et al. [3] succinctly show the transformation of the square law that applies for the long channel to the linear law that is applicable for short-channel MOSFET. On the other hand, *I-V* formulation for the CNTFET model follows the quantum conductance principle that was developed by Rahman et al. [4,5] and Datta [6]. The *I-V* model can be rewritten in terms of drain voltage *V*_d_, source voltage *V*_s_, and gate voltage *V*_G_ that is expressed by

(7) $$I_{\text{ds}}\left(V_{\text{G}},V_{\text{d}},V_{\text{s}}\right)=G_{\text{ON}}\frac{k_{\text{B}}T}{q}\left[\log\left(1+\exp\left(q\left(E_{\text{F}}-V_{\text{sc}}\left(V_{\text{G}},V_{\text{d}},V_{\text{s}}\right)\right)/k_{\text{B}}T\right)\right)\right]-G_{\text{ON}}\frac{k_{\text{B}}T}{q}\left[\log\left(1+\exp\left(q\left(E_{\text{F}}-V_{\text{sc}}\left(V_{\text{G}},V_{\text{d}},V_{\text{s}}\right)-V_{\text{d}}-V_{\text{s}}\right)/k_{\text{B}}T\right)\right)\right]\text{,}$$

where *G*_ON_ is the ON-conductance, *V*_sc_ is also known as the channel surface potential [11], *E*_F_ is the Fermi energy, *k*_B_ is the Boltzmann constant, *T* is the temperature, and *q* is the electric charge. The equation is iteratively solved and hence includes the effect of gate voltage.

### Model verification

**Figure 4:**
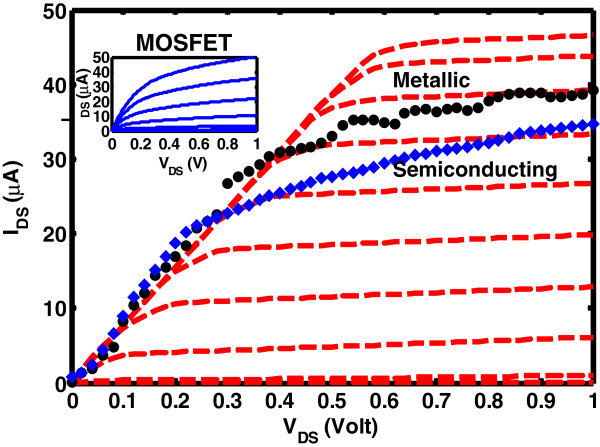
** *I* - *V* characteristic of SWCNT model, semiconducting and metallic CNT experimental data.***IV* characteristic of a 50-nm SWCNT model (dotted lines) demonstrated in comparison to *L* ≈ 50 nm semiconducting CNT experimental data (filled diamond). Metallic CNT experimental data are also shown (filled circle). Inset shows 45-nm MOSFET characteristics where the dimension is given in Table 2. Initial *V*_G_ at the top for CNT and MOSFET is 1 V with 0.1-V steps. (Adapted from [10]).

In this section, the potential of CNT circuit design is assessed. Our simulation results in Figure 4 indicate that CNTFET is able to provide drain current performance comparable to a 45-nm-gate length MOSFET. The model is successful in predicting expected output current levels in a sub-100-nm-channel CNT transistor experimental data. The DIBL effects and subthreshold swing (SS) are better suppressed in the CNT device, while the Si transistor demonstrates a moderate DIBL and SS due to short-channel effects as shown in Table 2. Although the CNT has similar ON-current, it sustains *I*_on_/*I*_off_ ratio of two orders of magnitude lower than Si MOSFET. The quantum ON-conductance limit of a ballistic single-walled carbon nanotube (SWCNT) and graphene nanoribbon with perfect contact is *G*_ON_ = 4*e*^2^/*h* and *G*_ON_ = 2*e*^2^/*h* (twice the fundamental quantum unit of conductance), respectively*.* Quantum capacitance *C*_Q_ is directly proportional to the density of states of the semiconductor but inversely proportional to the electrochemical potential energy. When *C*_Q_ becomes smaller than *C*_ox_, a large quantity of the electrochemical potential energy is needed to occupy the states above the Fermi energy. This results in the reduction in overall intrinsic gate capacitance *C*_G_ and limits the channel charge in a semiconductor and ultimately the *I*-*V* characteristic of the FET devices. Comparison in Table 2 shows that MOSFET has a higher cutoff frequency due to higher transconductance as compared to CNTFET with lower capacitances.

**Table 2:** **Device model specification at *V* **_**GS**_ **= 1 V**

**Parameter**	**CNTFET benchmarking**	
	**CNTFET**	**MOSFET**
Channel length, *L*	50 nm	45 nm
Contact width, *W*_contact_	100 nm	-
Channel width, *W*	-	125 nm
Channel area	5 × 10^−15^ m^2^	5.63 × 10^−15^ m^2^
Nanotube diameter	1.5437 nm	-
Chiral vector [**n**,**m**]	[20,0]	-
Maximum current, *I*_dmax_	46.56 μA	50.20 μA
Transconductance, *g*_m_	68.1 μS	148 μS
Carrier density, *I*_dmax_ / [*d* or *W*]	30.16 μA/nm	0.40 μA/nm
Gate capacitance, *C*_G_	14.85 aF	65.8 aF
Drain capacitance, *C*_d_	0.59 aF	19.0 aF
Source capacitance, *C*_s_	1.43 aF	78.7 aF
Substrate capacitance, *C*_sub_	1.60 aF	6.52 aF
Total terminal capacitance, *C*_ter_	18.47 aF	209.02 aF
Intrinsic capacitance, *C*_int_ *= C*_gd_ *+ C*_db_	21.29 aF	37.40 aF
Load capacitance, *C*_L_ at 1 GHz	46.54 fF	50.13 fF
Cutoff frequency with 5-μm wire	13.57 GHz	27.72 GHz
Drain-induced barrier lowering	40.85 mV/V	83.89 mV/V
Subthreshold swing	72.3 mV/decade	113.67 mV/decade
On-off ratio	2.99 × 10^4^	9.54 × 10^6^

First, MOSFET logic circuits are built based on a 45-nm generic PDK. The MOSFET designs are then compared with carbon-based circuit models that consist of prototype digital gates implemented in HSPICE circuit simulator. These CNTFETs use 45-nm process design rules, namely the minimum contact size. For a fair assessment, both MOSFET and CNTFET are designed to provide similar current strength (≈46 to 50 μA).

**Figure 5:**
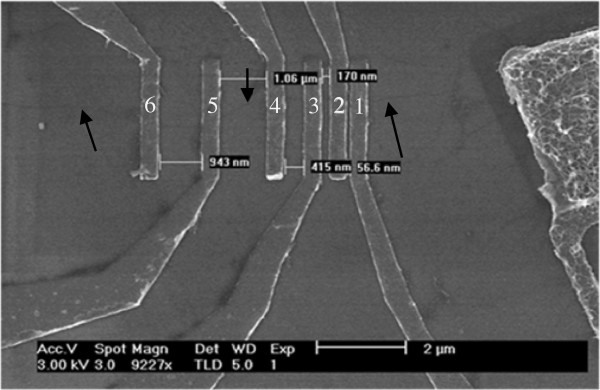
**Scanning electron microscope image of Pd contacts over the nanotube with each contact being labeled.** Black arrows are used to point to the SWCNT.

An appropriate CNTFET device was fabricated to investigate the contact resistance. SWCNTs were grown in situ using the bimetal catalyst iron-molybdenum (Fe-Mo) [11] on a silicon-on-insulator substrate with 200 nm of thermally grown SiO_2_. Metal contacts were patterned by electron beam lithography, and 60 nm of palladium (Pd) contacts was deposited to form a back gate geometry transistor. The spacing between the Pd contacts varied between 56.6 nm and 1.06 μm as shown in Figure 5.

A four-probe measurement was carried out at room temperature to extract the resistance characteristics of the carbon nanotube that was used to form the transistor channel. The normalized resistances were 0.495, 0.744, 0.118, and 0.450 MΩ/nm for *R*_2,3_, *R*_2,4_, *R*_3,4_, and *R*_4,5_, respectively, where indices indicate Pd contact labels. The diameter of the SWCNT is 1.5 nm. Calculation shows that the 415-nm nanotube resistance is 27.8 kΩ that is almost equal to the theoretical *R*_ON_ *= h/q*^2^ = 25.812 kΩ and four times larger than the theoretically lowest quantum resistance of the SWCNT, *R*_ON_ *= h/4q*^2^ = 6.5 kΩ.

Though at 415-nm channel length ballistic transport is not preserved in the CNT, it is still only factor 4 larger than the theoretically expected minimum, suggesting that scattering is not extensive. Nevertheless, the model which assumes ballistic transport predicts similar saturation current levels (≈50 μA) for both the 50- and 415-nm channel devices, as illustrated in Figure 5. Practically, this suggests that one must have CNT channel lengths below approximately 100 nm or even low contact resistance in order to utilize ballistic transport in them.

## Results and discussion

### Circuit analysis

**Figure 6:**
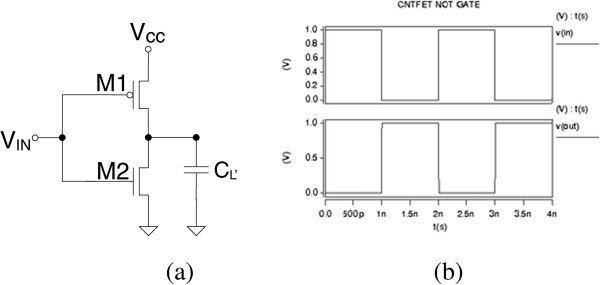
Schematic of NOT gate with parasitic capacitance (a); input and output waveforms for CNTFET (b).

**Figure 7:**
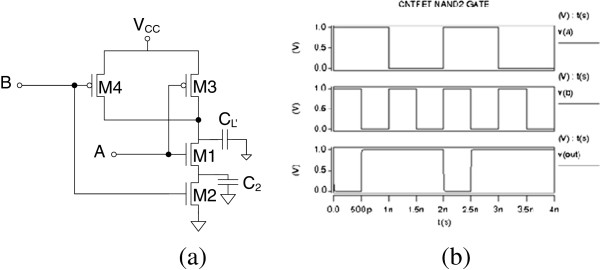
Schematic of two-input NAND2 gate with parasitic capacitance (a); input and output waveforms for CNTFET (b).

**Figure 8:**
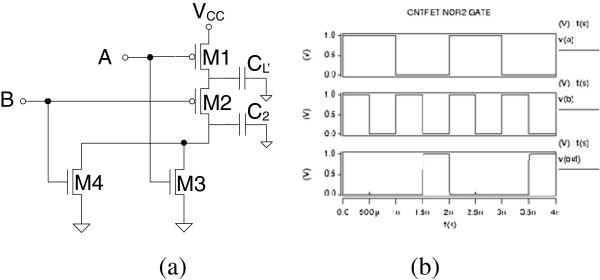
Schematic of two-input NOR2 gate with parasitic capacitance (a); input and output waveforms for CNTFET (b).

**Figure 9:**
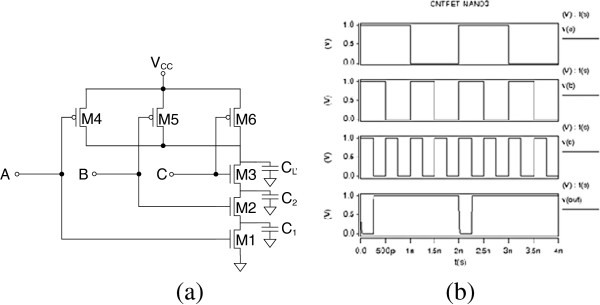
Schematic of three-input NAND3 gate with parasitic capacitance (a); input and output waveforms for CNTFET (b).

**Figure 10:**
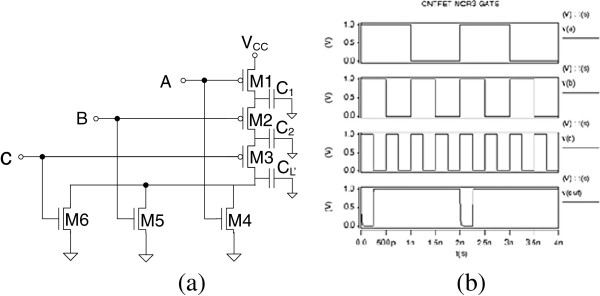
Schematic of three-input NOR3 gate with parasitic capacitance (a); input and output waveforms for CNTFET (b).

CNT circuit logic operation is simulated in HSPICE based on the compact models described in the ‘Model verification’ section. Figures 6, 7, 8, 9, and 10 show the schematic of NOT, NAND2, NOR2, NAND3, and NOR3 gates and their corresponding input and output waveform, respectively. It is shown that CNTFETs are able to provide correct logical operation as MOSFET from the output waveform. In this simulation, it is assumed that both the n-type and p-type CNTFETs have symmetrical *I*-*V* characteristics. The performance evaluation of these Boolean operations is listed in Table 3.

**Table 3:** 45-nm process propagation delay computation between CNTFET (with and without interconnect) and MOSFET (post-layout simulation)

**Logic circuits**	**CNTFET with 45-nm process design guidelines**		**MOSFET with 45-nm process**
	**Delay without interconnects**	**Delay with 5-μm interconnect**	**Delay (post-layout simulation)**
	**Propagation delay,**	**Propagation delay,**	**Propagation delay,**
	** *t* **_**p**_**(ps)**	** *t* **_**p**_**(ps)**	** *t* **_**p**_**(ps)**
NOT	0.14	9.277	5.005
NAND2	0.39	12.97	8.719
NAND3	0.61	16.87	11.343
NOR2	0.47	12.98	8.797
NOR3	0.50	16.48	11.655

### Performance evaluation

**Figure 11:**
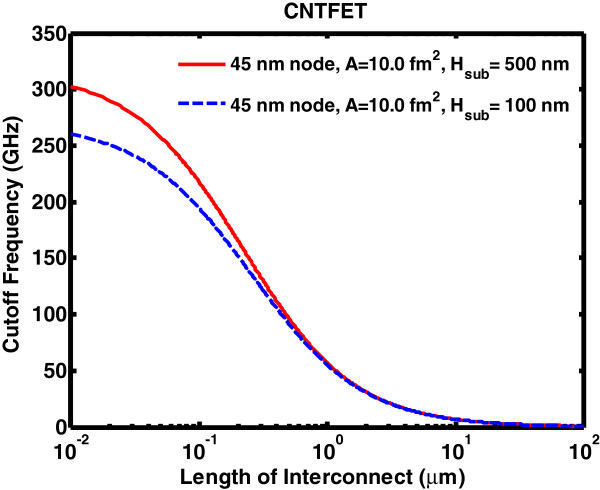
**Cutoff frequency for CNTFET.** Cutoff frequency for CNTFET with interconnect length from 0.01 to 100 μm with a source and drain contact area equivalent to that of a 45-nm MOSFET and substrate insulator thickness of 100-nm and 500-nm.

The unity current gain cutoff frequency for the CNTFET circuit model is depicted in Figure 11. The model uses a copper interconnect of 45 nm with a 100-nm and 500-nm substrate insulator thickness. The interconnect length varies from 0.01 to 100 μm. The length of interconnects affects considerably the frequency response. The lower length interconnect enhances the cutoff frequency. The substrate thickness also plays an active role in lower length domain. No distinction with the substrate thickness is visible beyond 1-μm interconnect length. The figure of merit for logic devices, namely power-delay product (PDP) and energy-delay product (EDP) metrics, are given as

(8) $$\text{PDP}=P_{\text{av}}\times t_{\text{p}}\text{;}$$

(9) $$\text{EDP}=\text{PDP}\times t_{\text{p}}\text{,}$$

where *P*_av_ is the average power and *t*_p_ is the propagation delay.

**Figure 12:**
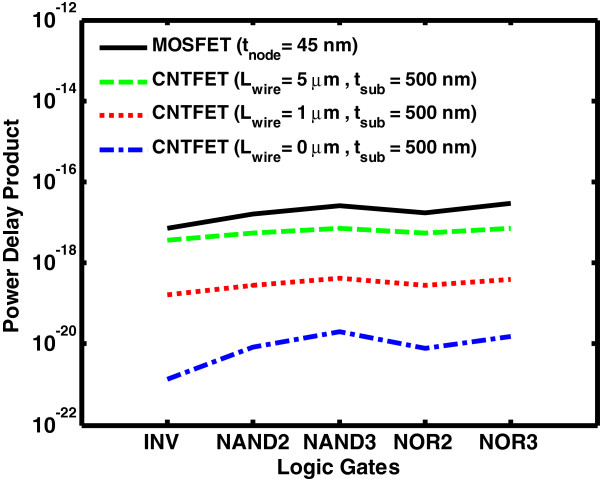
PDP of CNTFET versus MOSFET.

Figure 12 shows the PDP of CNTFET and MOSFET logic gates for the 45-nm process. The simulation results show that the PDP of CNTFET-based gates are lower than that of MOSFET-based gates by several orders of magnitude [18]. For the 45-nm process, the PDP of CNTFET-based gates is two times smaller than that of MOSFET-based gates with *L*_wire_ = 5 μm. It increases to 1,000 times without interconnect (*L*_wire_ = 0 μm).

**Figure 13:**
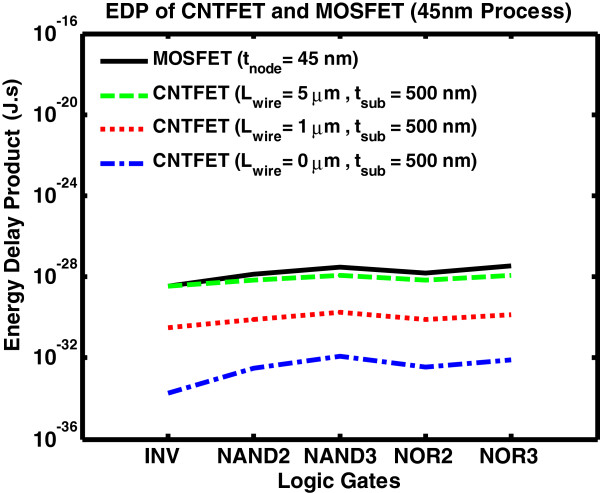
EDP of CNTFET versus MOSFET.

Figure 13 shows the EDP of CNTFET and MOSFET logic gates for the 45-nm process. EDP for CNTFET-based gates with 5 μm is comparable to MOSFET. As a result, the wire length should be kept shorter than 5 μm in order to obtain energy-efficient low-power architecture.

**Figure 14:**
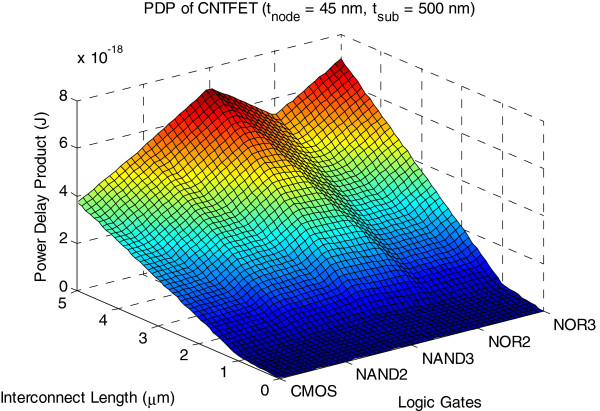
**3D plot of PDP of CNTFET logic gates.** The copper interconnect length is up to 5 μm for *t*_node_ = 45 nm and *t*_sub_ = 500 nm.

**Figure 15:**
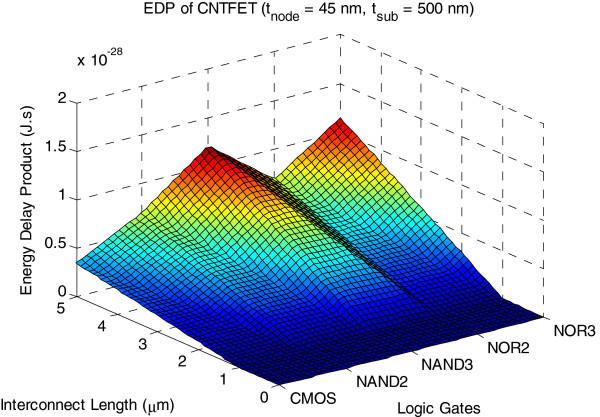
**3D plot of EDP of CNTFET logic gates.** The copper interconnect length up to 5 μm for *t*_node_ = 45 nm and *t*_sub_ = 500 nm.

Figures 14 and 15 show 3D plots of PDP and EDP for CNTFET with copper interconnect up to 5 μm in length. We observe a 28 % improvement of PDP while EDP reduces by 39 % for NAND3 that adopts the 45-nm process compared to the one that uses the 90-nm process contact size.

Table 3 shows the average propagation delay, *t*_p_, for logic gates NOT, NAND2, NAND3, NOR2, and NOR3 for CNTFET with and without interconnect in comparison with MOSFET during post-layout simulation. It is found that NAND3 or NOR3 has the largest propagation delay since both of them has multiple fan-in and fan-out each. In the digital logic simulation of CNTFET, we use an average length of 5 μm per fan-out.

## Conclusions

We have established that a longer channel CNT is capable of delivering output currents comparable to those from a 45-nm-node Si MOSFET. This is possible due to the preservation of ballistic transport over distances approaching 100 nm and the higher current density of a single CNT forming the channel. Consequently, in the same practical channel area, a CNT allows reduction of short-channel effects as it has a lower *E*_max_, leading to a lower DIBL and off current.

Devices with thicker substrate insulator and smaller source drain contact area give the highest frequency. In addition to that, logic gates NOT, NAND2, NAND3, NOR2, and NOR3 and their corresponding input and output waveforms are given. The interconnect length of cascading logic gates has a profound effect on the signal propagation delay. In the digital logic simulation, the key limiting factor for high-speed CNT-based chips is the interconnect itself. The performance enhancement of these carbon-based material is negligible if the interconnect capacitance is not reduced significantly with transistor feature size. Bundled metallic MWCNTs are seen as a potential candidate to replace copper interconnects as future IC interconnects once the challenges of integrating CNT interconnects onto existing manufacturing processes are met.

We also show that ballistic transport is not maintained in a CNT when contact resistance is large. A good fit to the data output characteristics from a 50-nm CNT channel device is obtained. As mean free path in a CNT is very long, often exceeding 1 μm, the ballistic process plays a predominant role, similar to one discussed extensively by Riyadi and Arora [19]. In fact, they define a new feature, named ballisticity. The truly ballistic transport is possible as channel length approaches zero. In a finite length, there are always finite probabilities of scattering.

## Competing interests

The authors declare that they have no competing interests.

## Authors’ contributions

MLPT designed and carried out the device modeling and simulation work, analyzed the data, and drafted the manuscript. GL carried out the experimental work and fabricated the SWCNT. GAJA supervised the research work and helped amend the manuscript. All authors read and approved the final manuscript.

## Authors’ informations

MLPT was born in Bukit Mertajam, Penang, Malaysia, in 1981. He received his B. Eng. (electrical-telecommunication) and M. Eng. (electrical) degrees from Universiti Teknologi Malaysia (UTM), Skudai, Malaysia, in 2003 and 2006, respectively. He conducted his postgraduate research in nanoscale MOSFET modeling at the Intel Penang Design Center, Penang, Malaysia. He recently obtained his Ph.D. degree in 2011 at the University of Cambridge, Cambridge, UK. He is a senior lecturer at UTM. His present research interests are in device modeling and circuit simulation of carbon nanotube, graphene nanoribbon, and MOSFET. MLPT is an IEEE member, member of IET (MIET), graduate member of IEM (GRAD IEM), and member of Queens' College. GL was born in Chania, Crete, Greece in 1983. He holds a B. Eng. (computing and robotic systems) degree from the Department of Electric Engineering in Liverpool University and a Ph.D. degree in engineering from the University of Cambridge. His Ph.D. thesis was in the area of fabricating and characterizing single-walled carbon nanotubes and ZnO nanowire transistors and sensors. He has also worked as a researcher at Nokia's Eurolab between 2009 and 2011 and particularly in developing novel sensors as part of Nokia's Nanosensing group. He, as part of Cambridge-M.I.T i-Teams, examines, identifies, and analyzes commercial potentials for an Intelligent Textbook technology, which uses an artificial intelligence engine, with real target customers in relevant industries. At present, GL is interested in pursuing a career that combines technology and analytical expertise, veiled in a business management environment. He is a member of Churchill College. GAJA received his B.Sc. degree in electrical/electronic engineering from Cardiff University, Wales, UK, in 1979 and his Ph.D. degree in electrical/electronic engineering from the University of Cambridge, Cambridge, UK, in 1983. He has held the 1966 Professorship in Engineering with the University of Cambridge since 1998. He currently heads the Electronics, Power and Energy Conversion Group, one of four major research groups within the Electrical Engineering Division of the Cambridge Engineering Faculty. He has worked for 25 years on integrated and discrete electronic devices for power conversion and on the science and technology of carbon-based electronics for 22 years. He has an active research program on the synthesis and electronic applications of carbon nanotubes and other nanoscale materials. He also has research interest in nanomagnetic materials for spin transport devices. He currently sits on the steering committee of the Nokia-Cambridge University Strategic Collaboration on Nanoscience and Nanotechnology and is the head of the Nokia-CU Nanotechnology for Energy Programme. His current research is focused on integrated power conversion circuits. He has previously held faculty positions at the University of Liverpool (Chair in Electrical Engineering), University of Cambridge, and University of Southampton. He has held the UK Royal Academy of Engineering Overseas Research Award at Stanford University, Stanford, CA, USA, and been a Royal Society visitor at the School of Physics, University of Sydney, Sydney, New South Wales, Australia. He has published over 450 journal and conference papers. GAJA was elected a Fellow of the Royal Academy of Engineering in 2004. In 2007, he was awarded the Royal Academy of Engineering Silver Medal ‘for outstanding personal contributions to British engineering.’
